# Effects of Atmospheric Plasma Corona Discharges on Soil Bacteria Viability

**DOI:** 10.3390/microorganisms8050704

**Published:** 2020-05-11

**Authors:** Yulia Lazra, Irina Dubrovin, Victor Multanen, Edward Bormashenko, Yelena Bormashenko, Rivka Cahan

**Affiliations:** Department of Chemical Engineering and Biotechnology, Ariel University, Ariel 40700, Israel; Yulia.Lazra@gmail.com (Y.L.); Irinadu@ariel.ac.il (I.D.); V.g.multanen@gmail.com (V.M.); Edward@ariel.ac.il (E.B.); yelenabo@ariel.ac.il (Y.B.)

**Keywords:** corona plasma discharge, bacteria, soil disinfection, bacterial relative distribution

## Abstract

Crop contamination by soil-borne pathogenic microorganisms often leads to serious infection outbreaks. Plant protection requires disinfection of agricultural lands. The chemical and the physical disinfection procedures have several disadvantages, including an irreversible change in the soil ecosystem. Plasma, the “fourth state of matter” is defined as an ionized gas containing an equal number of negatively and positively charged particles. Cold-plasma technology with air or oxygen as the working gas generates reactive oxygen species, which are found to efficiently eradicate bacteria. In this study, we examined the effect of atmospheric plasma corona discharges on soil bacteria viability. Soil that was exposed to plasma for 60 s resulted in bacterial reduction by two orders of magnitude, from 1.1 × 10^5^ to 2.3 × 10^3^ cells g^−1^ soil. Exposure for a longer period of 5 min did not lead to further significant reduction in bacterial concentration (a final reduction of only 2.5 orders of magnitude). The bacterial viability was evaluated using a colorimetric assay based on the bacterial hydrogenases immediately after exposure and at selected times during 24 h. The result showed no recovery in the bacterial viability. Plasma discharged directly on bacteria that were isolated from the soil resulted in a reduction by four orders of magnitude in the bacterial concentration compared to untreated isolated bacteria: 2.6 × 10^−3^ and 1.7 × 10^−7^, respectively. The plasma-resistant bacteria were found to be related to the taxonomic phylum *Firmicutes* (98.5%) and comprised the taxonomic orders *Bacillales* (95%) and *Clostridiales* (2%). To our knowledge, this is the first study of soil bacteria eradication using plasma corona discharges.

## 1. Introduction

Contamination of vegetables by fecal pathogenic bacteria is a severe problem that occurs from fertilizing soil with organic matter, such as poultry manure and cow dung. In addition, in arid areas, the scarcity of freshwater resources increases the demand for the use of treated wastewater in agriculture. The use of organic fertilizer and/or treated wastewater in agriculture leads to serious infection outbreaks, especially diarrhea [1,2]. Crop losses caused by soil-borne pathogenic microorganisms, animals, pests, and weeds affect about 20–40% of global agricultural productivity [3]. Plant protection includes disinfection methods that are based on chemical and physical approaches. The chemical methods include directly adding toxic chemical reagents, such as chloroform, ethylene oxide, bromomethane, hydrogen peroxide, and mercuric chloride [4]. Although the chemical reagents kill pathogenic bacteria, fungi, and pests living in the soil, they carry numerous disadvantages. The chemicals are absorbed into crops, and the residue from the reagents can also pollute soil and water sources [5]. Several disinfection chemicals, such as chloropicrin, are considered carcinogenic, and the fungicide methyl bromide causes a depletion of the earth’s ozone layer [6,7]. In addition, the chemical reagents change the microbial soil ecosystem and diminish the beneficial bacteria required for soil fertilization and pollutant bioremediation [5,8,9]. Physical methods consist of covering the soil with plastic sheets in the hot season, preventing use of the land for about two months. In addition, some of the physical methods consume high amounts of energy [10].

Nevertheless, improvement of crop quality and yields requires disinfection of agricultural lands. Given the above disadvantages of the common chemical and physical disinfection procedures, new approaches are needed to develop soil disinfection that can provide alternatives. In the current study, a new approach using atmospheric cold plasma was demonstrated.

Plasma, the “fourth state of matter”, is defined as an ionized gas containing an equal number of negatively and positively charged particles. Plasma can be classified according to the temperature of the so-called “cold” and “hot” discharges. In the cold plasmas, the temperature of heavy particles is relatively low compared to the high temperature of electrons, whereas particles constituting hot plasma are in thermal equilibrium. Low-temperature plasma can be further classified by the gas pressure. Plasma generated under atmospheric pressure is considered “atmospheric-pressure plasma”, whereas plasma discharges created under low vacuum (0.1–0.5 Torr) are regarded as “low-pressure plasma discharges” [11].

Cold-plasma technology is already applied in a variety of processes, including modification of the surface properties of organic and synthetic materials [12], bacterial deactivation in sewage sludge [13], wastewater treatment [14], biofilm formation on plasma-treated wood waste for bioremediation of toxic pollutants [15], biofilm formation on plasma-treated carbon-cloth anodes in microbial electrolysis cells for hydrogen formation [16], and advanced treatment of agriculture seeds [17].

Cold atmospheric-pressure plasma has become an attractive technology for microbial inactivation in the food industry and agricultural production, since the microorganism eradication occurs at low temperatures. Plasma sterilization efficiency depends on power input, gas composition, mode of exposure, and bacterial species [18,19,20]. Higher voltage of the plasma system and extended treatment duration increase the sterilization efficiency [19,20,21]. Bacterial species found to be sensitive to plasma treatment are both Gram-positive and Gram-negative, including *Staphylococcus aureus* [19], *Escherichia coli*, *Listeria monocytogenes* [20], *Pseudomonas aeruginosa* [21], and *Legionella pneumophila* [22]. It was shown that exposure of broccoli seed surfaces to a corona discharge plasma jet for 3 min reduced the contaminating microorganisms in the range of 1.2–2.3 log units [23]. Exposure of seeds to low pressure plasma for 10 min reduced the survival rate of the artificial contaminant pathogenic fungus, *Rhizooctonia solni,* to 1.7% without significant effects on the germination rate [24]. Low-pressure plasma treatment (voltage was 5.5 kV, argon gas flow rate was 0.5 L min^−1^) of *Xanthomonas campestris* (6.6 log colony-forming units (CFU) per seed) led to a decrease of these cruciferous seed-plant pathogenic bacteria by 3.9 log after 5 min of exposure and by 6.6 log after 40 min [25].

The plasma’s antimicrobial effect and mechanism include the formation of large quantities of reactive oxygen species (ROS) in the water phase, including hydroxyl radicals (•OH), ozone (O_3_), atomic oxygen (O), hydrogen peroxide (H_2_O_2_), and singlet oxygen (^1^O_2_), which were reported to damage the bacterial structure and functions [26,27,28]. In addition, the multiple reactive nitrogen species (RNS), including nitric oxide (NO), peroxinitrites (ONOO^−^), nitrites (NO^2−^), and nitrates (NO^3−^), play a major role in the plasma’s biocidal process by altering the cell wall components, the functions and the structure of the phospholipid bilayer, the structure of nucleic acids and cellular proteins, gene expressions, and protein synthesis [29,30,31]. Plasma treatment was shown to affect bacterial membranes. Plasma treatment (20 min) led to a decrease in the membrane integrity of *S. aureus* and *E. coli* to percentages of 18.54 ± 3.13% and 10.08 ± 2.50%, respectively. In addition, there was a decrease in the concentration of the bacterial cellular proteins from 47.33 ± 1.56 μg·mL^−1^ to 8.52 ± 1.02 μg·mL^−1^ for *S. aureus,* and 50.21 ± 2.22 μg·mL^−1^ to 6.31 ± 0.73 μg·mL^−1^ for *E. coli*. The cellular nucleic acid concentration was decreased for both bacteria. For *S. aureus*, the percentage decreased to 57.14 ± 4.34%, and for *E. coli*, it was reduced to 32.35 ± 2.82% [28].

Although plasma technology is known for its ability to eradicate microorganisms in general, there is limited information about the effect of plasma on soil microorganisms. Further study is needed to make plasma technology applicable for soil sterilization.

In this study, soil was exposed to plasma corona discharges for 15–60 s and for a longer period of 10 min. The bacterial eradication level was evaluated using a viable count assay by calculating the CFU g^−1^ soil and with a colorimetric assay based on the microorganism hydrogenases. The bacteria that were resistant to plasma were examined using 16S rRNA. To our knowledge, this is the first study of soil bacteria eradication using plasma corona discharge.

## 2. Materials and Methods

### 2.1. Plasma Corona Discharge System and Experimental Conditions

**Figure 1:**
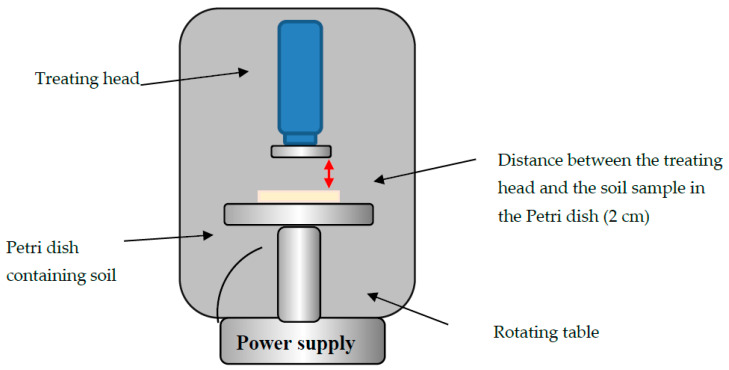
Sketch of the atmospheric corona discharge device (plasma device) and the rotating table.

Our plasma corona discharge device (3DT, MULTIDYNE 1000, Germantown, WI, USA) consisted of a treating head that contained two hook-shaped wire electrodes. The plasma was generated under high voltage at electrode 2 × 12 kV and a frequency of 50 Hz at atmospheric pressure conditions, using ambient air as a carrier gas. A rotating table (15 × 15 cm) made of steel covered by a thick layer of PVC was placed under the treating head. A power supply (PowerPac^tm^ basic, Bio-Rad, Hercules, CA, USA) was attached to the table, allowing the upper part of the table to rotate at a desired number of rounds per min. A Petri dish containing the soil sample (3–5 replicates) or the isolated bacteria eluted from the soil (3–5 replicates) was placed in the center of the rotating table. The distance between the sample and the treating head was adjusted to 2 cm. The corona discharge device scheme is illustrated in Figure 1.

### 2.2. Soil Exposure to Corona Discharge

Hamra soil was sifted in a strainer (pores of 1 × 1 mm) immediately before each experiment. A thin layer of soil sample (5 g) was placed in a Petri dish and mixed with phosphate-buffered saline solution (PBS, 2 mL) to prevent soil dispersal (by air currents from the plasma fan). Then, it was exposed to plasma for 15–60 s. When the soil was treated for a longer period of 5 min, it was exposed in a cycle regimen (60 s of exposure alternating with 60 s of rest). During each rest period, the soil was mixed with 2 mL PBS. The experiments were carried out under ambient conditions.

### 2.3. Viable Count Assay

#### 2.3.1. Measurement of Bacterial Concentration in Soil as a Function of Plasma Treatment

The plasma-treated soil (5 g) was collected from the Petri dish in PBS (10 mL) and transferred to a sterile tube. The soil suspension (100 μL) was serially diluted, and the appropriate dilutions were pour-plated on Luria-Bertani (LB) agar plates followed by incubation at 30 °C for 72 h. Viable bacterial cells were determined by counting the CFU and multiplying it by the corresponding dilutions. The results of the CFU were calculated for 1 g soil. The same procedure was performed for the control sample, excluding exposure to plasma.

#### 2.3.2. Measuring the Concentration of Isolated Soil Bacteria as a Function of Plasma Treatment

Preparation of the soil bacterial suspension was as follows: soil (5 g) was suspended in 10 mL PBS, vigorously vortexed, and incubated at 30 °C for 30 min while letting the soil settle into sediment. The supernatant (100 μL) was spread on LB agar and incubated at 30 °C for 72 h. All the colonies were collected and suspended in PBS, followed by dilution to 0.1 OD 590 nm. Then, 1 mL of the bacterial suspension (0.1 OD 590 nm) was centrifuged for 5 min at 10,000 rpm. The bacterial sediment was suspended in 100 μL PBS, from which a layer (1 × 1 cm) was spread on a Petri dish. This soil bacterial layer was exposed directly to plasma for 1 min.

The treated bacteria were collected in 1 mL PBS, transferred to an Eppendorf tube, and vortexed. Then, 100 μL of the suspension was serially diluted, and the appropriate dilutions were spread on LB agar plates and incubated at 30 °C for 72 h. Viable cells were determined by counting the CFU and multiplying them by the corresponding dilutions. The results of the CFU were calculated for 1 mL PBS.

### 2.4. Measurements of Soil Bacterial Viability

Measurement of the soil bacterial viability was based on the reduction of a tetrazolium-salts reagent by bacterial hydrogenases activity. This reaction results in a purple solution of varying intensity, which can be measured spectrophotometrically. Soil that was exposed to plasma for 5 min (pulse of 60 s alternating with 60 s of rest) was collected from the Petri dish using 10 mL PBS. The soil suspension was centrifuged at 3500× *g* for 5 min. The supernatant was decanted, and 7 mL of 3-(4,5-dimethylthiazol-2-yl)-2,5-diphenyltetrazolium bromide (MTT) solution (5 mg mL^−1^ of MTT in 0.1 M PBS, pH 7.4) was added to the soil sediment and left undisturbed for 2 h. The soil suspended in the MTT solution was centrifuged (3500× *g* for 5 min), and the reduced MTT salts attached to the soil sediment were dissolved by adding 7 mL dimethyl sulfoxide (DMSO): EtOH (1:1) for 20 min. The absorbance of the solution was examined using a spectrophotometer at 540 nm [15]. When the absorbance was higher than 1 OD, the sample was diluted and re-examined.

### 2.5. Examination of the Relative Bacterial Population Distribution as a Function of Plasma Treatment

A soil sample (5 g) was placed on a Petri dish and treated with plasma for 1 min. The soil was collected in 10 mL PBS, seeded on a LB agar dish, and incubated at 30 °C for 72 h. All the colonies were collected, and the bacterial population was identified and characterized by 16S rRNA analysis (Next Generation Sequencing, Hy-Labs, Rehovot, Israel).

DNA was extracted using the DNeasy Powersoil kit (Qiagen, Hilden, Germany) according to the manufacturer’s instructions. The 16s amplicon libraries were generated using a two-step PCR protocol. The V4 region of the 16s rRNA gene was amplified in the first PCR using the primers 515F and 806R [32] with the following change; instead of the Golay sequences, the 16s primers had tails CS1 and CS2 (Fluidigm, San Francisco, CA, USA). The second PCR was performed on the first PCR product using the Fluidigm Access Array primers for Illumina (FLuidigm, San Francisco, CA, USA) to add the adaptor and index sequences required for Illumina sequencing. Sequencing was conducted on the Illumina Miseq, using a v2–500 cycles kit to generate 2 × 250 paired-end readings. Demultiplexing was performed on Base Space (the Illumina cloud) to generate FASTQ files for each sample. The data were furthered analyzed based on CLC-bio (Aarhus, Denmark) to generate operational taxonomic unit (OTU) and abundance tables.

### 2.6. Statistics

Values are presented as means ± SEM. Statistical differences between the treatments and the controls were tested by unpaired two-tailed Student’s *t*-test or one-way analysis of variance (ANOVA) followed by Bonferroni’s post-hoc testing when appropriate. A difference of *p* < 0.05 or less in the mean values was considered statistically significant.

## 3. Results and Discussion

### 3.1. Bacterial Soil Concentration as a Function of Plasma Corona Discharge Exposure Duration and Rotation Rate

**Figure 2:**
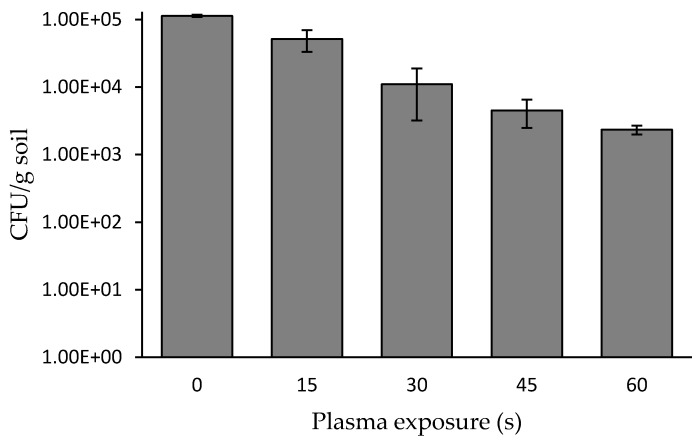
Bacterial concentration in one-gram soil samples as a function of plasma exposure duration. A one-way ANOVA followed by Bonferroni’s multiple comparison post-hoc test was conducted to compare the effect of plasma exposure duration (nontreated (0 s), 15, 30, 45, 60, and 90 s) on bacterial concentration. Post-hoc comparisons using the Bonferroni adjustment indicated that mean score of nontreated bacteria (0 s) was significantly higher than all the other treatment conditions *(p* < 0.001). In addition, the analysis yielded that the 15 s condition was significantly higher than the 30, 45, 60, and 90 s conditions (*p* < 0.001). There were no other significant effects.

Soil (5 g) was spread to a homogeneous layer in a Petri dish followed by exposure to a plasma corona discharge for 15 to 60 s. Since the plasma treating-head exit size was 1 × 2 cm, and the distance between the treating head and the Petri dish was 2 cm, the Petri dish was rotated in order to expose the entire soil sample area. In this experiment, the Petri dish was rotated at 11 rounds per minute. A control sample of soil was treated in the same manner, excluding exposure to plasma. At the indicated times, the plasma-treated and untreated soils were collated in PBS, and 100 µL from each supernatant were spread on LB agar. After 72 h of incubation, the CFU per gram of soil was calculated for each exposure duration (Figure 2). The results indicated that plasma treatment for 30 s led to a bacterial reduction by one order of magnitude, from 1.1 × 10^5^ to 1.1 × 10^4^. Treatment for 60 s led to a bacterial reduction by two orders of magnitude, from 1.1 × 10^5^ to 2.3 × 10^3^ cells g^−1^ soil. In the experiment where the soil was exposed to plasma for a longer period (10 min) with the cycle regime described in Materials and Methods, there was no further significant reduction of the soil bacterial concentration, only resulting in a final reduction of 2.5 orders of magnitude.

**Figure 3:**
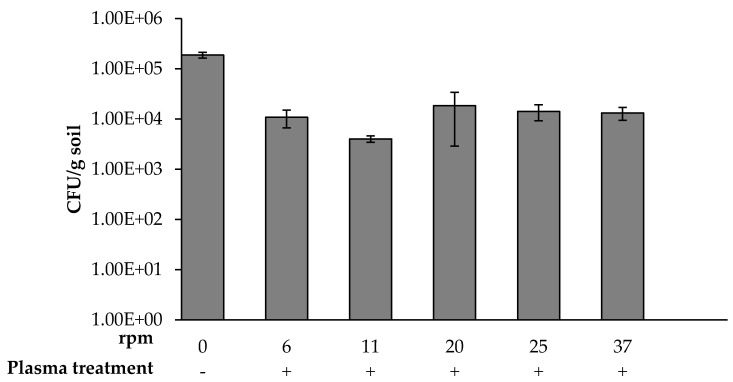
Bacterial concentration as a function of soil sample rotation rate (rpm) during 60 s of plasma exposure. A one-way ANOVA followed by Bonferroni’s multiple comparison post-hoc test was conducted to compare the effect of soil sample rotation rate (rpm) (0, 6, 11, 20, 25, and 37 rpm) on bacterial concentration. Post-hoc comparisons using the Bonferroni adjustment indicated that the mean score of 0 rpm was significantly higher than all the other rotation rate (*p* < 0.001). There were no other significant effects.

The rotation rate for maximal bacterial eradication during exposure to 60 s of plasma was determined by rotating the table containing the soil sample at varying rates of 6, 11, 20, 25, and 37 rpm. Each soil sample was collected, and the CFU per one gram was calculated (Figure 3). It was found that exposing the soil sample at 11 rpm decreased the bacterial concentration by about two orders of magnitude. In contrast, 6, 20, 25, and 37 rpm decreased the bacterial concentration by about one to 1.2 orders of magnitude. The D-value (decimal reduction time) required to kill 90% (or one log) of the soil bacteria using plasma treatment is about 30 s.

The antimicrobial effect of plasma was found to be due to the charged particles, electrons, reactive species, and UV that are found in a gas discharge [33]. The plasma working gas influenced the generation of both the amount and the type of the reactive species [34]. Oxygen and air are the most investigated gases for plasma bacterial sterilization. The reactive oxygen species (ROS) ^•^OH, ^•^O_2_^−^, ^1^O_2_, O_3_, and H_2_O_2_ are known to exert a major influence on the sterilization process. It was shown that plasma treatment led to a high level of intracellular ROS generation in *E. coli* and *S. aureus*. The ROS were found to affect either the bacterial cell envelope or the DNA. The level of damage and the affected site were correlated to the bacterial species [18]. The ROS were also found to cause a shrinking and etching of the plasma-treated bacterial spores [35]. Use of a metal mesh to separate charged particles from the other plasma agents revealed that charged particles led to disruption of the bacterial outer membrane followed by bacterial death [36].

### 3.2. Investigating Possible Recovery of Bacterial Soil Exposed to Plasma Corona Discharges

**Figure 4:**
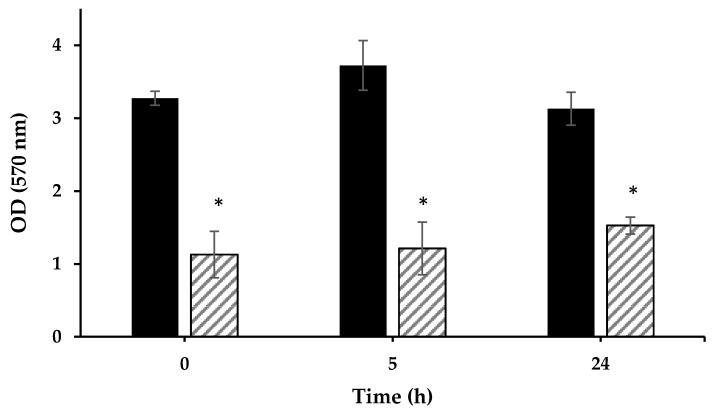
Bacterial viability using 3-(4,5-dimethylthiazol-2-yl)-2,5-diphenyltetrazolium bromide (MTT) analysis of soil that was exposed to plasma corona discharge for 5 min (60 s of exposure alternated by 60 s of rest, repeated five times). Soil that was exposed to plasma ( 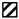 ), and the control sample not exposed to plasma ( 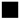 ). * *p* < 0.005 by Student’s *t*-test.

The soil was exposed to plasma corona discharge for 10 min, as described in Materials and Methods. The bacterial viability was examined by colorimetric assay immediately after exposure to plasma and again after 5 and 24 h. Since only some of the soil bacteria can grow on LB medium, the bacterial viability was also examined by the MTT reagent, a method that includes many other bacteria living in the soil that cannot grow on LB agar. Viability is revealed in a reduction of MTT by the hydrogenases of the bacteria. The reduced MTT is dissolved by DMSO:EtOH, and the intensity of the obtained solution is measured using a spectrophotometer (Figure 4). Examination of the soil bacterial viability showed that exposure for 5 min led to a three-fold reduction, which was maintained for 5 and 24 h, indicating that there was not a recovery in the bacterial viability during the 24 h of measurements.

### 3.3. The Concentration of Isolated Soil Bacteria as a Function of Plasma Corona Discharge Exposure

We assumed that the soil particles inhibit the full exposure of the bacteria to the plasma. Thus, plasma was applied directly on the detached bacteria from the soil. A soil sample was suspended in PBS followed by vigorous shaking using a vortex. The sample was incubated while letting the soil settle as sediment. The supernatant (100 µL) with the free bacteria was spread on LB agar followed by collecting all the colonies in PBS (final bacterial turbidity was 0.1 OD 590 nm). The bacterial suspension was centrifuged, and the bacterial sediment was spread on a Petri dish, as described in Methods. The bacterial layer was then exposed to plasma for 60 and 120 s. The plasma-treated bacteria were collected, and the CFU mL^−1^ was calculated. The same procedure was followed for a control sample, excluding exposure to plasma.

**Figure 5:**
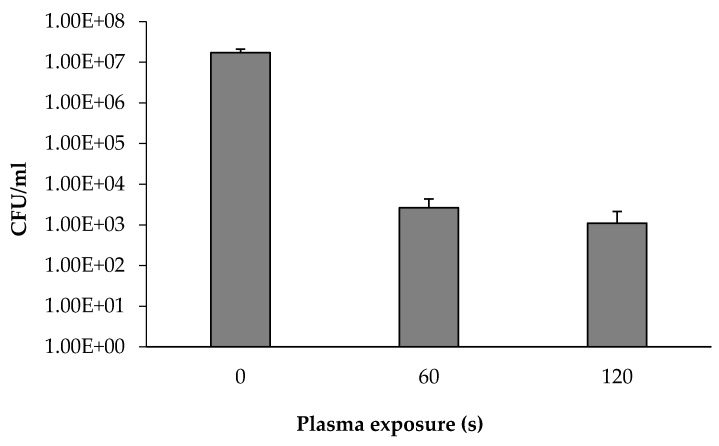
Isolated soil bacteria concentration as a function of plasma exposure duration. A one-way ANOVA followed by Bonferroni’s multiple comparison post-hoc test was conducted to compare the effect of plasma exposure duration (nontreated (0 s), 60, and 120 s) on isolated soil bacterial concentration. Post-hoc comparisons using the Bonferroni adjustment indicated that mean score of the nontreated (0 s) was significantly higher than all the other treatment conditions (*p* < 0.001). There were no other significant effects.

As depicted in Figure 5, plasma treatment conducted directly on the isolated soil bacteria for 60 s resulted in a decrease by four orders of magnitude in the bacterial concentration, compared to untreated detached bacteria: 2.6 × 10^−3^ and 1.7 × 10^−7^, respectively. Longer plasma exposure (120 s) did not lead to a further decrease in the bacterial concentration.

Nonthermal plasma treatment for bacterial decontamination has been reported for Gram-positive, Gram-negative, and biofilm-forming bacteria [33,37,38]. That bacterial inactivation was applied in the open air at atmospheric pressure or in a vacuum chamber instrument [39,40]. The inactivation efficacy of atmospheric cold plasma against *Staphylococcus aureus* Gram-positive and *E. coli* Gram-negative bacteria was found to be correlated with treatment time. The treated Gram-positive bacteria was mainly inactivated by intracellular damage, while the Gram-negative bacteria expired mainly by cell leakage [18]. Xu. et al. showed that plasma treatment led to damage of the bacterial cell wall of both *E. coli* and *S. aureus* and a decrease in the total concentrations of nucleic acid and cellular protein. However, *S. aureus* was less susceptible to plasma exposure in comparison to *E. coli* [28].

Bacterial eradication via plasma technology was found to be influenced by environmental factors, such as pH, humidity, and the matrix on which the bacteria were placed during plasma exposure [14]. Kayes et al. showed a reduction of 4.9 log when *Bacillus cereus* was treated at pH 5, while a reduction of only 2.1 log was observed at pH 7 [41]. Humidity was also reported as an important parameter; increasing the relative humidity was correlated to efficiency in plasma inactivation of *Aspergillus niger,* which was explained by the generation of more hydroxyl radicals [42]. Regarding the matrix on which the bacteria were placed during plasma treatment, higher eradication was observed when microorganisms were loaded on a filter compared to a fruit surface [43]. Additionally, higher eradication efficacy of *Salmonella Typhimurium*, *E. coli,* and *Listeria monocytogenes* was observed on agar plates compared to sliced cheddar cheese [44]. The antibacterial efficiency of a plasma reactor based on ambient air was examined on several bacterial strains. A significant inhibition of *E. coli* and *S. epidermidis* was observed within 1 min of application. The pH and the temperature were not changed after the plasma exposure revealed that neither changes of pH nor heat played any role in the bacterial inactivation. The antibacterial effect on *S. aureus* that was seeded on several surface types such as agar and plastics (polypropylene or acrylonitrile butadiene styrene discs) showed a considerable variation depending on the surface tested. The surface moisture also influenced the inactivation level [45]. The fact that plasma’s bactericidal effect is influenced by environmental factors constitutes one of the disadvantages of this technology. Thus, for each environmental condition, all the physical parameters should be examined.

In addition, from our experiments, there is an influence by the substratum type on which the bacteria are placed and the increase of temperature during exposure to plasma. For example, when the soil or the isolated bacteria were placed on a Pyrex Petri dish, the temperature increased to about 60 °C. Thus, in this case, the high temperature may influence the bacterial disinfection rather than the exposure to plasma. However, when the samples were placed on a plastic Petri dish, the temperature reached only 35 °C).

Another disadvantage is that the plasma may penetrate in the liquid phase to only 2.5 ± 1 nm [46].

Soil disinfection is an important goal for increasing crop yield and quality. However, soil as a matrix for plasma disinfection has not been widely studied. Previously, it was shown that exposure of soil to a dielectric barrier reactor with a high-voltage electrode and air or oxygen as the gas flow led to reduction in soil bacteria by two orders of magnitude, from 3.8 × 10^7^ to 8.5 × 10^5^. Dielectric barrier discharge plasma with air or oxygen as the gas flow did not change the soil mineral content; however, the plasma treatment reduced the pH from 6.2 before treatment to six afterwards [4].

### 3.4. Relative Distribution of the Soil Bacterial Population as a Function of Plasma Treatment

**Figure 6:**
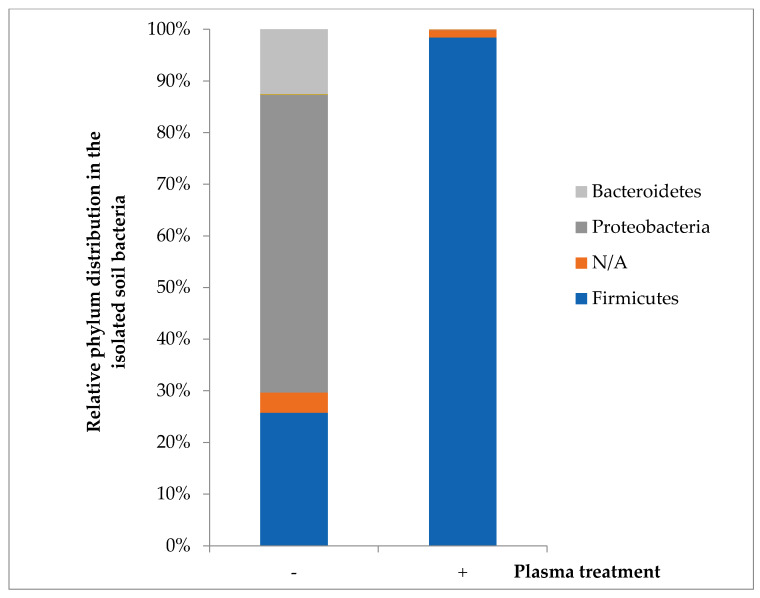
Relative distribution at the phylum level of the bacteria from the plasma-treated soil (+) and nontreated soil (−).

A soil sample placed in a Petri dish and was exposed to plasma for 60 s. The sample was collected in PBS and spread on agar plates followed by incubation for 72 h at 30 °C. All the colonies from each agar plate were collected, and the bacterial population was identified and characterized by 16S rRNA gene sequencing analysis (Figure 6). The microbial diversity in the plasma-treated and untreated soils was likewise evaluated based on 16S rRNA. Operational taxonomic unit (OTU) reads were identified and phylogenetically classified.

In the bacteria from the nontreated soil, the predominant phylum was *Proteobacteria* (56%), mainly divided into the taxonomic orders *Pseudomonadales* (42%) and *Xanthomonadales* (13%). The second-most predominant was *Firmicutes* (25%), mainly divided into the orders *Bacillales* (17%) and *Clostridiales* (4%). The third-most predominant was *Bacteroidetes* (12%), mainly divided into the orders *Sphingobacteriales* (8%) and *Flavobacteriales* (4%). The other phyla (5%) each gave evidence of relatively low presence. It is important to note a group of unassigned phyla (4%), which may be attributed to either a significant amount of novel species or poorly identified taxonomy.

In the bacteria from the plasma-treated soil, the phylum distribution was significantly different. The predominant phylum was *Firmicutes* (98.5%) with a small number of unassigned phyla (1.4%). The other identified phyla (2) comprised less than 0.1%. The *Firmicutes* phylum mainly divided into the taxonomic orders *Bacillales* (95%) and *Clostridiales* (2%);

To summarize, 98.5% of the bacteria surviving the plasma treatment belonged to the orders *Bacillales* and *Clostridiales*, many members of which are spore-forming bacteria [47]. In the control sample, the relative distribution of *Bacillales* and *Clostridiales* was only 21%.

The plasma resistant bacteria were spread on LB agar. The most abundant colony was related to the spore-forming bacteria *Bacillus* sp. One colony was collected and inoculated in a fresh LB broth as well as LB agar and incubated at 36 °C for 72 h. About 90% of the sample were spores. Bacterial suspensions (0.1 OD, 1 mL) of the LB broth as well as from the LB agar were washed and suspended in PBS. The samples (0.05 mL) were exposed to plasma for 60 s. The same procedure was done to the control sample (nontreated bacteria) except for exposure to plasma. The samples were collected with PBS, and the viable bacterial concentration was examined. The results showed that there was not a significant effect between the plasma-treated and nontreated bacteria (about 10^7^ CFU mL^−1^). This phenomenon indicates that *Bacillus* sp. spores are resistant to plasma treatment of the described regime.

According to prokaryote genome sequences, there are four abundant bacterial phyla: *Proteobacteria, Firmicutes*, *Actinobacteria,* and *Bacteroidetes,* out of 35 phylum-level bacteria [47]. The bacteria in *Firmicutes* are mesophilic, thermophilic, or psychrotrophic; anaerobic or aerobic and use organic molecules or minerals for ATP formation. This phylum includes the orders *Bacillales* and *Clostridiales,* many members of which can sporulate in response to harsh environmental conditions, such as nutritional limitation or high cellular density [48,49,50]. The genetic material inside the spore is also resistant to severe environmental conditions, such as desiccation, high temperature, caustic chemicals, and radiation. The resistance is triggered by the unique protective multilayered envelops, partial dehydration of the spore, calcium dipicolinic acid, and small acid-soluble proteins. The multilayer envelope is composed of an internal membrane, a cortex, an outer membrane, a coat, and an exosporium [51]. Each layer has a specific structure as well as biochemical and permeability properties. The spore structure is species-specific and influenced by the sporulation conditions [52]. Spore-forming *Bacillus subtilis* bacteria were exposed to atmospheric cold plasma applying a mixture of helium and oxygen or pure helium. Scanning electron microscopy (SEM) images illustrated morphological changes in the *Bacillus subtilis* spores after 5 min of treatment. Fluorescence images of the treated sample indicated severe spore damage. Exposing the *Bacillus subtilis* spores for 10 min to the atmospheric-helium plasma led to a four-log reduction in the live spores [53].

The intrinsic microbial characteristics play an important role in the efficiency of the sterilization. Tseng et al. showed that plasma treatment of both *E.* coli and vegetative cells of *B.* subtilis resulted in a decimal-value reduction in less than 0.5 min, while more than 2.5 min were required for spores [53]. The disinfection efficiency of plasma treatment on the Gram-negative bacteria (which possess an outer membrane and a thin layer of peptidoglycan) compared to the Gram-positive bacteria (having a thicker layer of peptidoglycan without outer membrane) was investigated with contradictory results. Gram-negative bacteria were mostly reported to be more sensitive to plasma than the Gram-positive [44,54]. For example, the D values needed to eradicate Gram-negative *E.* coli and *Salmonella* *Typhimurium* were 0.70 and 0.19 min, respectively, whereas for Gram-positive *Listeria* monocytogenes, it was 1.19 min [44]. Other studies revealed no differences between Gram-positive and Gram-negative bacteria in resistance to plasma [55]. The controversy could be explained by a longer exposure time and stronger intensity in the studies that showed the same lethal efficiency for both sorts of bacteria.

Eradication of soil-borne bacterial pathogens is complicated and is one of the major challenges in agriculture. For decades, a variety of chemicals were widely used for controlling pathogens. One of the most used chemicals is the methyl bromide, which was proved to be toxic to humans and the environment [56]. Soil solarization, which is a physical method, is based on the use of polyethylene sheets to cover the soil and capture heat during summer months. This method is considered effective without the adverse effect of chemicals [57]. The disadvantages of soil solarization technology are that it requires long times of application (about 50 days); in addition, it increased the abundance of heat-resistant bacteria [58].

In this study, an alternative approach using cold plasma technology is proposed for soil disinfection. Cold plasma is known as eco-friendly technology. The plasma’s antimicrobial effect and mechanism include the formation of ROS and RNS that alter the cell components, leading to bacterial death [29,30,31]. However, there are several disadvantages. The penetration of plasma was found to be in a very short distance; for example, in the liquid phase, it was only 2.5 ± 1 nm [46]. Additionally, full plasma exposure of bacteria that attached to soil particles may be limited. These two limitations may be solved by a vigorous mixing of the soil while exposing it to plasma. Another disadvantage shown in this study is that spore-forming bacteria are resistant to atmospheric plasma corona discharges. We assume that a combination of plasma treatment with a chemical reagent in low concentration may increase the spore disinfection efficiency.

Soil disinfection is an important goal for increasing crop yield and quality. Atmospheric plasma corona discharges may be an eco-friendly and cost-effective method alternative to the common chemical and soil solarization treatments. However, a comprehensive study on the effect of plasma treatment on the soil matrix such as mineral and pH should be explored.

## 4. Conclusions

The effects of atmospheric plasma corona discharges were examined on soil bacteria by exposing the soil to plasma for 30 and 60 s. These resulted in bacterial reduction by one order of magnitude (1.1 × 10^5^ to 1.1 × 10^4^) and about two orders of magnitude (1.1 × 10^5^ to 2.3 × 10^3^ cells g^−1^ soil), respectively. Exposure for a longer period of 10 min did not lead to further significant reduction in bacterial concentration (a total of only 2.5 orders of magnitude). The bacterial viability was evaluated by colorimetric assay (MTT analysis), which is based on the bacterial hydrogenases. The reduction of the bacterial viability of the treated soil was three-fold compared to the non-treated soil. Examination of bacterial viability within 24 h after plasma treatment showed no recovery in the bacterial viability. Plasma exposure directly on bacteria that were isolated from the soil resulted in a reduction by four orders of magnitude in the bacterial concentration compared to untreated isolated bacteria (2.6 × 10^−3^ and 1.7 × 10^−7^, respectively). The plasma-resistant bacteria were found to be related to the taxonomic phylum *Firmicutes* (98.5%) divided into the taxonomic orders *Bacillales* (95%) and *Clostridiales* (2%). In contrast, the non-treated bacteria included only 21% of *Bacillales* and *Clostridiales*. Eradication of soil bacteria was less effective than direct plasma treatment of isolated bacteria. Spore-forming bacteria were found to be resistant to atmospheric plasma corona discharges. We assume that spore eradication needs further investigation, which may include a combination of plasma treatment with a chemical reagent in low concentration. In addition, a vigorous mixing of the soil may expose more bacteria to the plasma.
